# DetoxiProt: an integrated database for detoxification proteins

**DOI:** 10.1186/1471-2164-12-S3-S2

**Published:** 2011-11-30

**Authors:** Zhen Yang, Ying Yu, Lei Yao, Guangui Li, Lin Wang, Yiyao Hu, Haibin Wei, Li Wang, Riadh Hammami, Roxanne Razavi, Yang Zhong, Xufang Liang

**Affiliations:** 1 School of Life Science, Fudan University, HanDan Road 220#, Shanghai, 200433, China; 2 College of Life Science and Technology, Jinan University, Guangzhou, 510632, China; 3 Key Laboratory of Nutrition and Metabolism, Institute for Nutritional Sciences, Shanghai Institutes for Biological Sciences, Chinese Academy of Sciences; Shanghai, 200031, China; 4 Shanghai Center for Bioinformation Technology, Shanghai, 200235, China; 5 Department of Food Sciences and Nutrition, Pavillon Paul-Comtois, Université Laval, 2425 rue de l'Agriculture, Québec, QC, Canada G1V 0A6; 6 Department of Biology, Queen's University, Kingston, Ontario, Canada K7L 3N6; 7 Institute of Biodiversity Science and Geobiology, Tibet University, Lhasa, 850000, China

## Abstract

**Background:**

Detoxification proteins are a class of proteins for degradation and/or elimination of endogenous and exogenous toxins or medicines, as well as reactive oxygen species (ROS) produced by these materials. Most of these proteins are generated as a response to the stimulation of toxins or medicines. They are essential for the clearance of harmful substances and for maintenance of physiological balance in organisms. Thus, it is important to collect and integrate information on detoxification proteins.

**Results:**

To store, retrieve and analyze the information related to their features and functions, we developed the DetoxiProt, a comprehensive database for annotation of these proteins. This database provides detailed introductions about different classes of the detoxification proteins. Extensive annotations of these proteins, including sequences, structures, features, inducers, inhibitors, substrates, chromosomal location, functional domains as well as physiological-biochemical properties were generated. Furthermore, pre-computed BLAST results, multiple sequence alignments and evolutionary trees for detoxification proteins are also provided for evolutionary study of conserved function and pathways. The current version of DetoxiProt contains 5956 protein entries distributed in 628 organisms. An easy to use web interface was designed, so that annotations about each detoxification protein can be retrieved by browsing with a specific method or by searching with different criteria.

**Conclusions:**

DetoxiProt provides an effective and efficient way of accessing the detoxification protein sequences and other high-quality information. This database would be a valuable source for toxicologists, pharmacologists and medicinal chemists. DetoxiProt database is freely available at http://lifecenter.sgst.cn/detoxiprot/.

## Background

Living organisms are exposed to the external environment and are continuously confronted with a variety of chemical compounds and xenobiotics. Many of these naturally occurring substances or man-made chemicals can be deleterious when inhaled or absorbed, or even hazardous to organisms, (e.g. plant toxins to prevent herbivory). Artificial chemicals include anti-cancer drugs, antibiotics produced by fungi, insecticide, herbicide and other environmental pollutants [1]. Some kinds of toxins can lead the cell to produce large amounts of reactive oxygen species (ROS), though in some cases, ROS can be natural by-products of metabolic processes. ROS can react with many cellular molecules and cause damage or contribute to the development of various pathologies [2]. Thus, self-protective mechanisms including catalytic biotransformation have evolved as an adaptation against various toxic chemical species. Cells possess various kinds of detoxification proteins that are capable of metabolizing a wide variety of toxins, mainly classified into Phase I and Phase II xenobiotic-metabolizing enzymes (XEMs) as well as antioxidant enzymes according to their functional mechanisms [3-5]. These proteins react directly with toxins to reduce their toxicity and enhance their solubility so that they can be easily eliminated from the cell.

Phase I reactions include oxidation, reduction and hydrolysis. A major function of the Phase I reaction is the transformation of substrates so that they can be easily eliminated or modified by reactive groups for subsequent Phase II reaction. The most important Phase I system is the cytochrome P450 enzyme (CYP). It is an important class of heme-containing monooxigenase that mainly catalyzes the oxidative reactions of the adjunction of an atom from molecular oxygen into a substrate. This superfamily can be functionally classified into three groups, with more than 50 protein members have been identified in humans [6]. The genetic association between CYP superfamily and various diseases has been identified, such as cancer, fatty liver disease and Parkinson's disease [7-9]. Phase II reactions include glutathione (GSH) conjugation, glucuronidation, sulfation, acetylation, and methylation. Small polar molecules, such as glutathione, UDP-glucuronic acid (UDPGA) and acetyl coenzyme A (AcCoA), can be conjugated with the toxic compounds [10-12]. Glutathione conjugation is the primary Phase II reaction. The glutathione S-transferase (GST) catalyzes the conjugation of the thiol group of GSH, the tripeptide gamma-Glu-Cys-Gly, with a wide variety of electrophiles. It has been widely demonstrated that the polymorphism of GSTs is related to cancer susceptibility and patient survival [13]. ROS produced by exogenous toxins or generated during the endogenous metabolic process can react with the oxidation enzymes and was eventually reduced by non-enzymatic antioxidant defenses, such as ascorbic acid (vitamin C), alpha-tocopherol (vitamin E) and GSH, or enzymatic antioxidant defenses, such as catalase (CAT), peroxidase (POD) and superoxide dismutase (SOD) [14,15]. Relatively high levels of oxygen in the brain make it one of the vulnerable organs to ROS damage. Many neurodegenerative diseases are associated with oxidative damage such as Parkinson's disease [16]. Thus, these proteins may constitute the major protective mechanism of the brain from damage due to oxidative stress [17].

Detoxification proteins have been found from invertebrates to vertebrates. More than one hundred genes encoding these proteins have been identified in humans. Large efforts have been made to understand the structural, functional and evolutionary basis of detoxification proteins, as well as their roles in disease development [18-20]. Many detoxification protein-related databases have been established to provide this kind of information, such as PeroxisomeDB [21] and PeroxiBase [22]. These databases mainly focus on peroxidase related proteins, which constitute only part of the steps in cellular detoxification. There is currently no system for the classification and annotation of all these proteins. Thus, the DetoxiProt database was created to facilitate the efficient annotation and acquisition of information about the detoxification proteins. The aim of the DetoxiProt is to provide a useful platform for detoxification protein researchers in the fields of physiology, pharmacology, toxicology, food security, farm chemical development and environmental pollution related research. It provides relevant information for the function and evolutionary analysis of these proteins. In addition to the chemical features of the genes, the DetoxiProt features 3D structures, sub-cellular location, tissue expression and especially, the inducer, inhibitor and substrates of these enzymes. Presently, the database contains 5956 entries encompassing 20 different detoxification protein families.

## Construction and content

**Figure 1:**
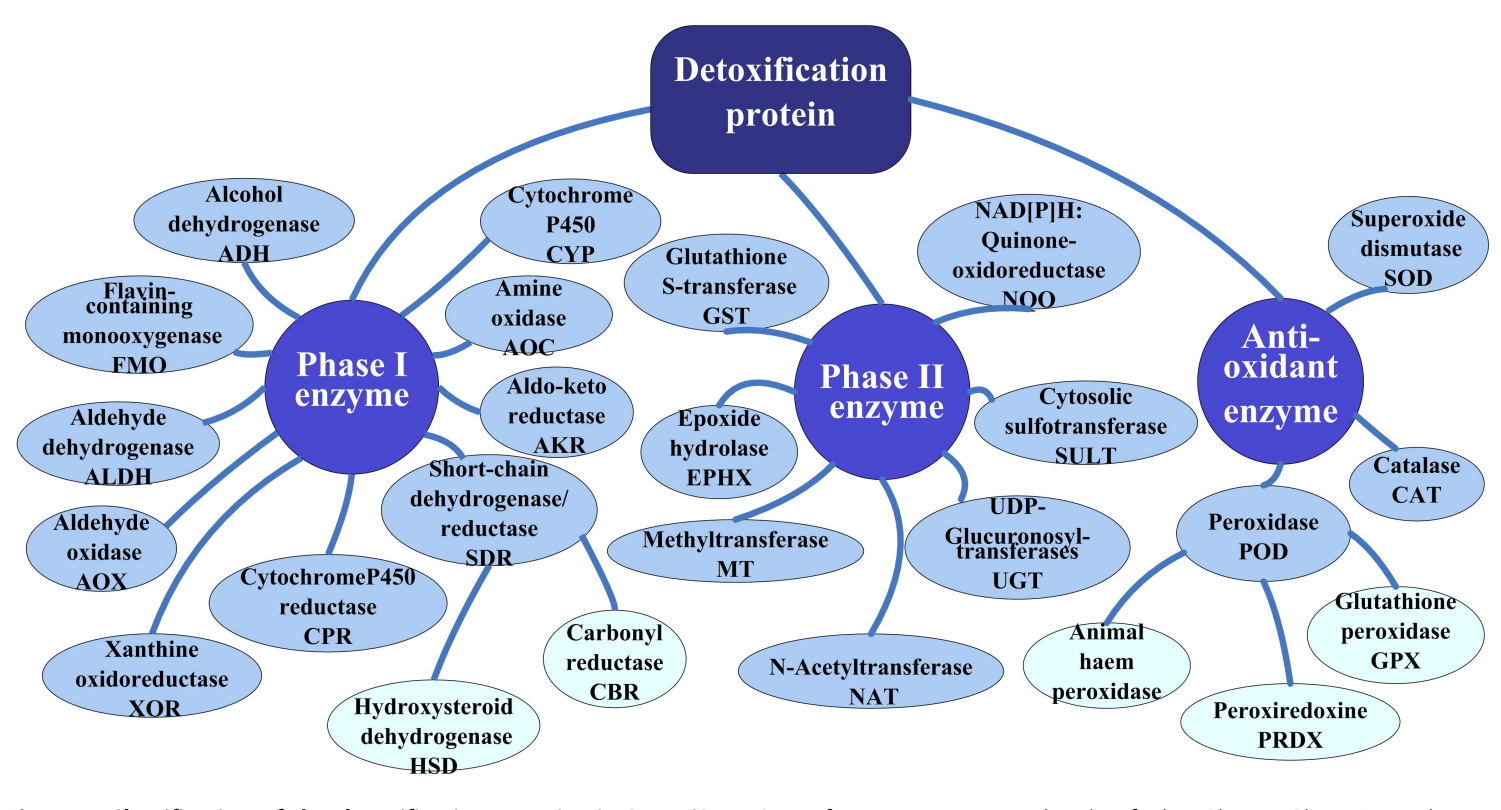
**Classification of the detoxification proteins in DetoxiProt.** Detoxification proteins can be classified as Phase I, Phase II xenobiotic-metabolizing enzymes and antioxidant enzymes according to their functional mechanisms. Totally 20 protein families were identified. Some protein families can be classified as subfamilies, including the short-chain dehydrogenase/reductase and peroxidase.

We extensively searched various public databases and related literature to collect information on detoxification proteins. Data collection was performed by the following approaches: we first performed a systemic search for relevant literature and determined the precise classification of these proteins. Accordingly, 20 protein families were identified (Figure 1). For each protein family, a summary description was compiled displaying the gene symbol name, InterPro database ID and the protein introduction [23]. Then, the Kyoto Encyclopedia of Genes and Genomes (KEGG) [24], the UniProt Knowledgebase (UniProtKB) [25] and NCBI gene database were inquired to identify all the members of each family. Other public databases including Ethanol-Related Gene Resource (ERGR) [26], Aldo-Keto Reductase (AKR) Superfamily homepage [27] and PeroxiBase [22] were subsequently searched for further confirmation and supplementation. Then we queried through a list of the protein names as the keywords against SwissProt and NCBI protein database for collection of protein sequences [28]. For the completeness of the database, we used the BLAST embedded in NCBI to search homolog sequences. The final set of detoxification proteins were manually compiled, with a total of 5956 proteins from 628 organisms collected.

Extra information from other databases and literature, as well as the predicted protein features were incorporated into each detoxification record. Protein cofactors, tissue distribution and cellular location were retrieved from the references and publicly available databases such as NCBI and UniProtKB, while experimentally determined protein structures were retrieved from protein databank (PDB) [29]. DetoxiProt also provides the predicted protein physiological-biochemical properties including the molecular weight, theoretical pI, aliphatic index and Grand average of hydropathicity (GRAVY), which computed by the ProtParam tool from the Expasy server (http://www.expasy.org/tools/protpar-ref.html). To provide fast protein classification, protein domains were predicted by various web servers including the Pfam [30], Prosite [31] and SMART [32]. External database links such as NCBI Protein, UNIPROT, KEGG etc, were also included.

**Figure 2:**
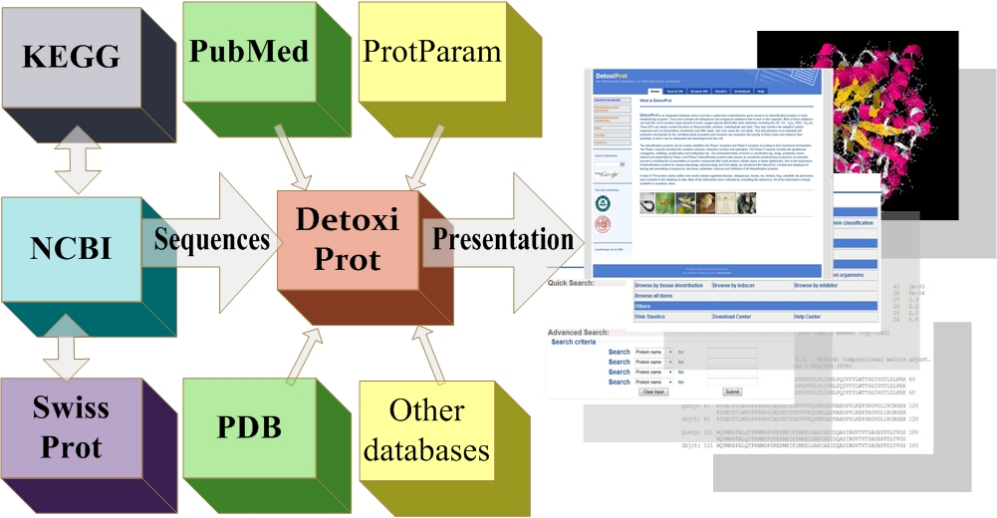
**Data source and integration of DetoxiProt.** Protein sequences are mainly sourced from NCBI and SwissProt, Protein structures were retrieved from PDB database, Biochemical feature were predicted by ProtParam tool from the Expasy server, substrates, inducers and inhibitors for detoxification proteins were manually compiled from literature.

Due to the importance of detoxification proteins for medicinal chemistry, we collected information from the literature the about the inducer, inhibitor and substrate for each protein from nine model organisms (*Caenorhabditis elegans*, *Danio rerio*, *Drosophila melanogaster*, *Gallus gallus*, *Homo sapiens*, *Mus musculus*, *Pan troglodytes*, *Rattus norvegicus* and *Xenopus laevis*). The nomenclatures of the toxins were revised and unified according to MeSH and PubChem [28]. Thus, each entry was manually annotated to ensure data quality. A detailed strategy for protein information collection and data integration can be seen in Figure 2.

### Database architecture

DetoxiProt was implemented as a relational database using MySQL. Five major tables were used to store data. Web interface was implemented using PHP language. The database is running on a Red Hat Enterprise Linux 3 with Apache as HTTP server (please see additional file 1).

## Utility

DetoxiProt provides a user-friendly interface allowing easy access to data. The “Home” page contains general information about the database and detoxification proteins. The “detoxification protein classification” page summarizes detailed information about the biological activity of each kind of protein. Other useful tools are also provided:

### Basic and advanced searches

The “Search DB” page permits users to perform a quick search and advanced search in the database. Gene symbol name, NCBI gene ID, UNIPROT ID and PDB ID are supported for the quick search. Advanced search tool allow users to combine multiple criteria including protein name, organism, tissue distribution, sub-cellular location, etc. This method helps users to locate specific proteins of interest rapidly and accurately. For example, a medicinal chemist or a pharmacologist may be particularly interested in the specific human cell membrane proteins that are inhibited by some kind of toxin, so the options of protein name, organism, cellular location and inhibitor in each of the pull-down lists can be selected separately and simultaneously, and records limited by specific keywords will be displayed.

### Peptide mapping and protein domain search

A tool of peptide mapping is designed for mapping the functional peptides to the entire sequence of detoxification protein. User defined peptide sequences can be exactly searched from protein sequences and the position information provided. This is helpful for understanding the distribution of the active peptides across entire proteins. Another useful tool to infer the functional diversity of detoxification protein is the protein domain search. The concept that conserved sequences among sequences may be a symbol of functionally similar protein domains underlies many classification methods of protein families. Thus we predict the domains within each protein by Pfam, Prosite and SMART web servers. A simple keyword search tool was provided to search database entries by domain or family name of interest to the user.

### Structural similarity search

DetoxiProt provides a structural similarity search tool, the 3D-BLAST [33,34], to search detoxification proteins that have similar 3D-structures. This method allows for a quick and accurate search based on protein structure. It identifies 23 states of structural alphabet, which are used to represent pattern profiles of the protein backbone fragment. Protein structures are represented as structural alphabet sequence databases (SADB). Users may upload their own structure files in PDB format, after which the 3D-Blast program uses BLAST to search for the longest common substructures, known as structural alphabet high-scoring segment pairs (SAHSP), between the structural alphabet sequence translated from query structure and that of other structures in the database. A statistical significance score (E-value) is calculated to indicate the reliability of the prediction of the alignment. Depiction of the results for different search methods is provided (please see additional file 2).

### Browse database

**Figure 3:**
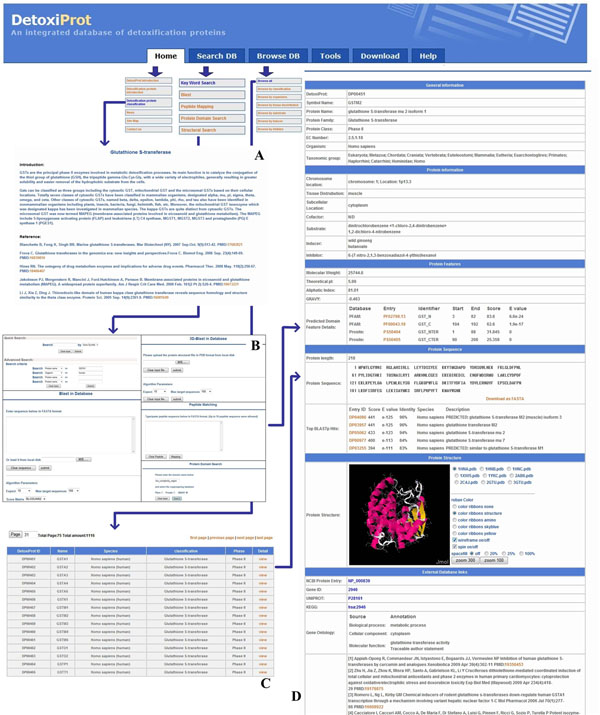
**DetoxiProt Interface.** (A) Description page for each protein class, (B) Protein query page of the database, (C) Bibliography browse page, (D) an example for search result of human GSTA1, protein entry DP00451, detailed information includes general information, protein features, protein sequences, protein structures and external database links.

Different browsing methods are implemented in the database to allow users to navigate by specific criteria. DetoxiProt provides seven different browsing methods: browse all data or specific dataset including the classification, species, tissue distribution, substrates, inducers and inhibitors. Cascading browse styles from specific dataset to protein list, then to detailed information are provided. The “detailed information” page includes general information, protein information, predicted protein features, protein structure, external database links and references. The 3D illustration of a protein structure may be viewed in interactive mode via the Jmol applet (http://www.jmol.org/) when the structure file is available. This tool would be particularly useful for protein structure investigation and drug development. While protein sequences may be individually download in FASTA format, the complete set of protein sequences may be retrieved from the “Download” page. Figure 3 shows an example of the detailed information on the “browse” page.

### Comparative genomics tools

A customized BLAST tool is embedded for sequence similarity search [35]. To identify potential paralogs or orthologs of each detoxification protein, pre-computed top-matched hits by BLAST search against other proteins in DetoxiProt were listed in the “detailed information” page. This would facilitate phylogenetic relationships studies of the genes across different organisms throughout evolution. Putative ortholog genes may be identified from the best reciprocal hits. For each of the nine model organisms, we generated multiple sequence alignments for each protein family by MUSCLE [36]. Corresponding phylogenetic trees were built by Maximum Likelihood (ML) method embedded in RAxML [37]. The nonparametric bootstrap test was performed for 100 replicates. A Java application of Archaeopteryx tree viewer is embedded for browsing and manipulation of the phylogenetic trees [38]. An example illustrating the result of cytosolic sulfotransferases of *Mus musculus* is available in the additional file 3. Both the multiple sequence alignment and phylogenetic tree pages can be found in the “tools” page.

## Discussion

Although information concerning specific types of detoxification proteins, for example, peroxidase or peroxisome related proteins, proteins involved in ethanol or drug metabolism, can be found in some databases including the PeroxisomeDB, PeroxiBase, ERGR and SuperTarget [39], a complete collection of all these categories of proteins is still deficient. Here, the key categories of proteins involved in detoxification are identified and classified. In addition, the targets identified for the detoxification proteins may provide clues to determine the possible effective enzymes in specific drug metabolism pathway. In total, 20 different protein families were identified to be involved in the functionality of detoxification after a literature review. For complete analysis of the database, we inspected the phylogenetic distribution of protein families across model organisms (please see additional file 4). All of the three major detoxification protein classes were found in the nine model organisms. For the protein families, relatively complete datasets were found in mammals. However, the aldehyde oxidases and xanthine oxidoreductases are missing in chimpanzees, the catalases are missing in chickens and seven protein families are missing in amphibia. In contrast, a relatively small number of proteins were found in invertebrates including the *Drosophila melanogaster* and *Caenorhabditis elegans*. The amount of detoxification genes took up about 1.5% of genomic genes in most model organisms. At the gene-family level, the cytochrome P450 family and the glutathione S-transferase family are the most abundant groups, with 68 and 48 members identified in humans, respectively. A relatively small number of proteins were found in non-model organisms, probably due to the incomplete annotation of the genes in these species.

**Figure 4:**
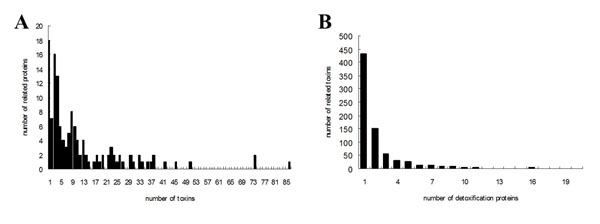
**Distribution of human detoxification proteins and related toxins documented in Detoxiprot.** (A) Histogram of the number of toxins associated with individual detoxification protein, (B) Histogram of the number of detoxification proteins associated with individual toxin.

In order to give an overview of the function of detoxification proteins, we collected all the 388 human genes and then explored their annotation of molecular function in Gene Ontology by means of the GOEAST [40]. Among 368 genes identified to have catalytic activities, more than 70% have the oxidoreductase activity, and about 30% have transferase activity and the function of ion binding. Other genes function for lipid binding, carboxylic acid binding and selenium binding (please see additional file 5). To provide an overview of protein and toxins relationships, we constructed a network to describe links of the relationship between human detoxification proteins and chemical compounds or environmental factors (here unified as toxins), which include the substrates, inducers and inhibitors compiled from the literature (please see additional file 6). Within this network, 18 detoxification proteins (13.6%) are associated with only one kind of toxin, while 78 detoxification proteins (59.1%) are associated with more than five kinds of toxins (Figure 4A). CYP1A1, CYP1A2, CYP3A4, CYP2B6 and UGT1A1 demonstrated the highest connectivity, which suggests the importance of the cytochrome P450 superfamily for elimination of toxins and maintenance of balance in the internal environment. CYPs represent the most important Phase I toxin/drug metabolizing enzymes. They are responsible for the metabolism of more than 80% of the clinical drugs. Inter-individual variation of CYP genes constitutes an important factor related to cancer susceptibility [41]. Similar to CYPs, The Phase II enzyme UGT is another important kind of drug metabolic protein. Association between UGT genes and diseases including cancer and fatty liver disease are already identified [42,43]. For the toxins, the metal ions, estradiol, tobacco smoke, polycyclic aromatic hydrocarbon and rifampin take the top five positions in our developed network. Figure 4B shows the number of proteins associated with toxins. 430 kinds of toxins (57.3%) were shown to be associated with only one protein. Although some bias may exist due to the literature investigated, nevertheless, this network provides a useful tool for inferring the relationships between detoxification protein and toxins.

## Conclusions

In summary, DetoxiProt is a powerful and reliable database that provides comprehensive information about detoxification proteins. Various browsing manners and powerful search engines were implemented providing users with useful ways to explore information in the database. We expect that DetoxiProt will serve as a useful tool for researchers in the detoxification protein field. It could also lead to better understanding of the function and evolution of detoxification proteins. The database will be continually updated, and as the increasing numbers of novel sequences become available, newly identified detoxification proteins will be added to the database. We also hope to adopt text-mining tools to help us to pre-screen protein-toxin relationships. These methods will continue to enhance the completeness of the database.

## Availability and requirements

The DetoxiProt may be accessed through http://lifecenter.sgst.cn/detoxiprot/. All the information is freely available to users. To browse the protein 3D structures and phylogenetic trees, the Java Runtime Environment (JRE) plug-in is required.

## Competing interests

The authors declare that they have no competing interests.

## Authors' contributions

XL and YZ conceived the concept of DetoxiProt. GL and LW collected and compiled data from literature and public databases. YY and ZY constructed the database and compiled the draft of the manuscript. LY incorporated protein structure manipulative tools for the database. YH, HW and LW participated in the design of the study and integrated protein data. RH and RR provided valuable suggestions and revised the manuscript. All authors read and approved the final manuscript.

## Supplementary Material

Additional file 1 **Database structure for DetoxiProt**. Five major table were used to store data, including the Main Info, predicted Domain, Mutual Blast result, Gene Ontology and Reference. Click here for file

Additional file 2 **Display of the results for different search method**. (A) Result for query result list, (B) Result for BLAST, (C) Result for peptide mapping, (D) Key word search result for protein domains, (E) Result for 3D-BLAST. Click here for file

Additional file 3 **Illustration of the phylogentic tree of Cytosolic sulfotransferases of *Mus musculus* by the Archaeopteryx tree viewer**. Multiple Sequence Alignment was performed by MUSCLE, phylogenetic tree was generated by Maximum Likelihood (ML) method embedded in RAxML. The nonparametric bootstrap test was performed for 100 replicates. Click here for file

Additional file 4 **Phylogenetic distribution of detoxification proteins in model organisms**. A relative small number of protein families were found in invertebrates than that in vertebrates. Click here for file

Additional file 5 **Gene ontology enrichment analysis of human detoxification genes**. This analysis was performed using GOEAST: http://omicslab.genetics.ac.cn/GOEAST/ Click here for file

Additional file 6 **A bipartite network demonstrating the relationship between detoxification proteins and related toxins**. This network comprises 1633 edges and 882 nodes. Red nodes represent detoxification proteins and green nodes are toxins. The nodes size represents degree of connectivity of the nodes. Key proteins (CYP1A1, CYP1A2, CYP3A4, CYP2B6 and UGT1A1) have highest degree of connectivity were labelled, with 86, 74, 74, 51 and 46 edges respectively. Click here for file
