# Supplementary figures and images for: DetoxiProt: an integrated database for detoxification proteins
Source: BMC Genomics. 2011 Nov 30;12(Suppl 3):S2. doi: 10.1186/1471-2164-12-S3-S2 (PMC3333179; doi:10.1186/1471-2164-12-S3-S2)

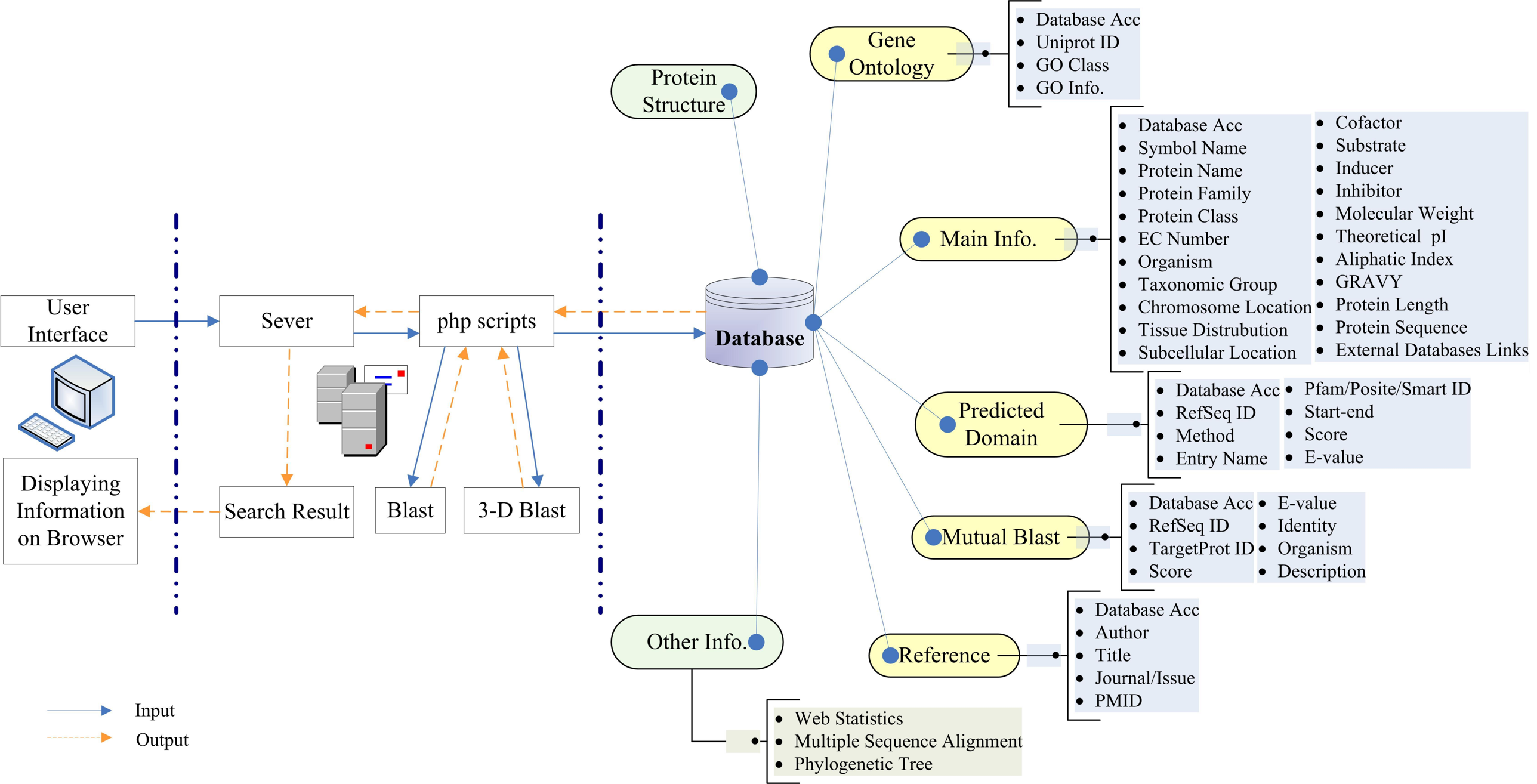

Supplement: Additional file 1 — Database structure for DetoxiProt. Five major table were used to store data, including the Main Info, predicted Domain, Mutual Blast result, Gene Ontology and Reference. [file 1471-2164-12-S3-S2-S1.pdf]

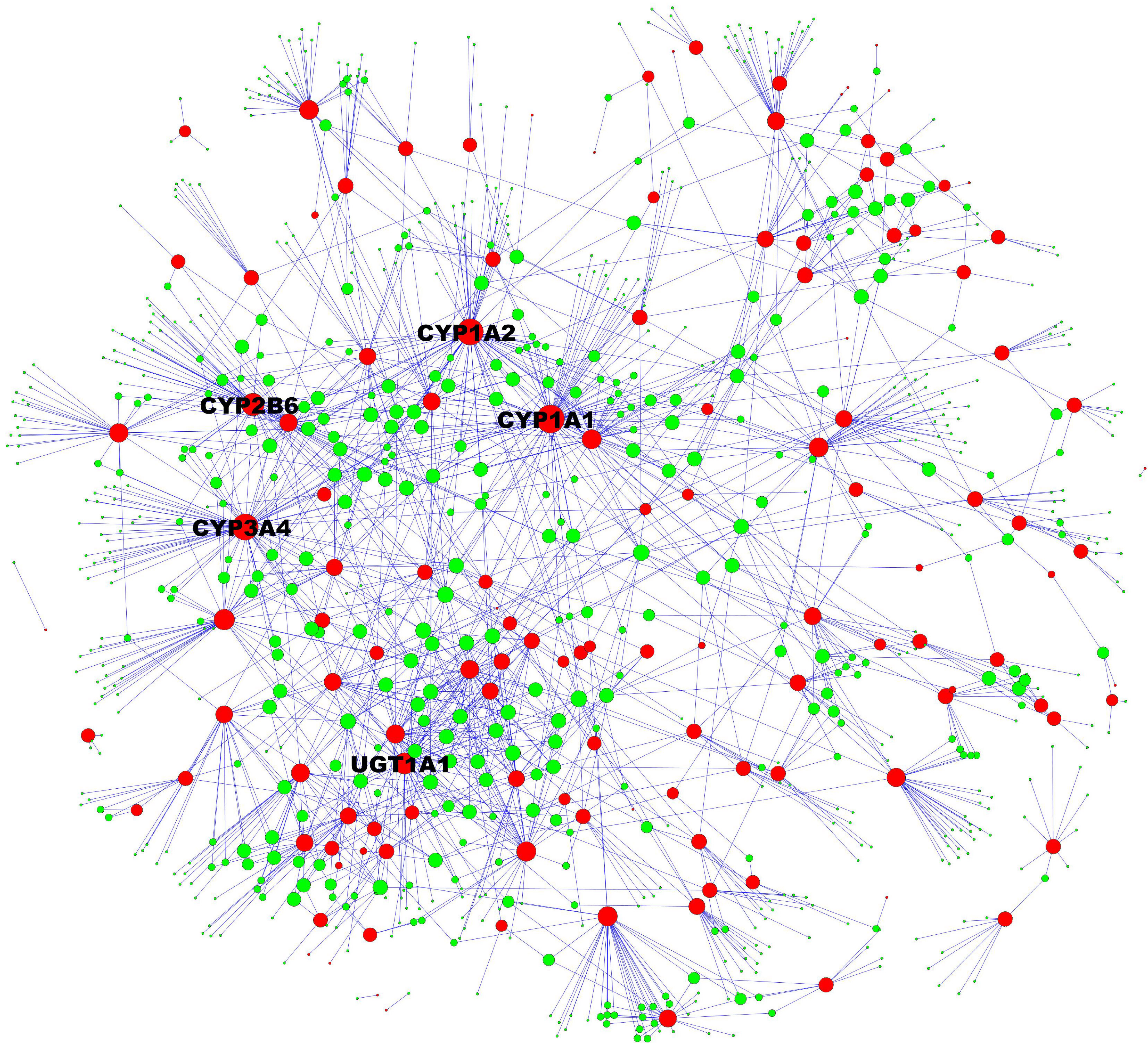

Supplement: Additional file 6 — A bipartite network demonstrating the relationship between detoxification proteins and related toxins. This network comprises 1633 edges and 882 nodes. Red nodes represent detoxification proteins and green nodes are toxins. The nodes size represents degree of connectivity of the nodes. Key proteins (CYP1A1, CYP1A2, CYP3A4, CYP2B6 and UGT1A1) have highest degree of connectivity were labelled, with 86, 74, 74, 51 and 46 edges respectively. [file 1471-2164-12-S3-S2-S6.pdf]
