# Supplementary material for: DetoxiProt: an integrated database for detoxification proteins
Source: BMC Genomics. 2011 Nov 30;12(Suppl 3):S2. doi: 10.1186/1471-2164-12-S3-S2 (PMC3333179; doi:10.1186/1471-2164-12-S3-S2)
Supplement: Additional file 2 — Display of the results for different search method. (A) Result for query result list, (B) Result for BLAST, (C) Result for peptide mapping, (D) Key word search result for protein domains, (E) Result for 3D-BLAST. [file 1471-2164-12-S3-S2-S2.pdf]

A

Query Result

DetoxiProt ID	Name	Species	classification	Phase	Detail
DP00104	ADH8A	Danio rerio	Alcohol dehydrogenase	Phase I	view
DP00105	ADH8B	Danio rerio	Alcohol dehydrogenase	Phase I	view
DP00116	MAO	Danio rerio	Amine oxidase	Phase I	view
DP00292	CYP1A1	Gallus gallus	Cytochrome P450	Phase I	view
DP00293	CYP1A4	Gallus gallus	Cytochrome P450	Phase I	view
DP00379	AKR7A2	Homo sapiens	Aldo-keto reductase	Phase I	view
DP00393	AOC3	Homo sapiens	Amine oxidase	Phase I	view

B

blast in Database

```
Sequences producing significant alignments:                    (bits) Value

DP00586  cytochrome P450, family 2, subfamily c, polypeptide...   595    e-171
DP00914  cytochrome P450, family 2, subfamily c, polypeptide...   456    e-129
DP00913  cytochrome P450, family 2, subfamily c, polypeptide...   428    e-121
DP00584  cytochrome P450, family 2, subfamily c, polypeptide...   409    e-115
DP00583  cytochrome P450, family 2, subfamily c, polypeptide...   408    e-115
DP00585  cytochrome P450, family 2, subfamily c, polypeptide...   401    e-113
DP00588  cytochrome P450, family 2, subfamily c, polypeptide...   399    e-112
DP00589  cytochrome P450, family 2, subfamily c, polypeptide...   390    e-109
DP00582  cytochrome P450, family 2, subfamily c, polypeptide...   387    e-109

 Query: 1   MAVESQXXXXXXXXXXXXXXXXXXSHAGKILLIPVDGSHWLSMLGAIQQLQQRGHEIVVL 60
             MAVESQ                  HAGK+LLIPVDGSHWLSMLG IQQLQQRGHEIVVL
 Sbjct: 1   MAVESQGRHPLVLGLLLCVLGPVLCHAGKMLLIPVDGSHWLSMLGTIQQLQQRGHEIVVL 60

 Query: 61  APDASLYIRDGAFYTLKTYPVPFQREDVKESFVSLGHNVFENDSFLQRVIKTYKKIKKDS 120
             APDASLYIR+GAFYTLKTYPVPFQREDVKESFVSLGHNVFENDSFL+RVIKTYKKIKKDS
 Sbjct: 61  APDASLYIREGAFYTLKTYPVPFQREDVKESFVSLGHNVFENDSFLRRVIKTYKKIKKDS 120

 Query: 121 AMLLSGCSHLLHNKELMASLAESSFDVMLTDPFLPCSPIVAQYLSLPTVFFLHALPCSLE 180
             AMLLSGCSHLLHNKELMASLAESSFDVMLTDPFLPC PIVAQYLSLPTVFFL+ALPCSLE
 Sbjct: 121 AMLLSGCSHLLHNKELMASLAESSFDVMLTDPFLPCGPIVAQYLSLPTVFFLNALPCSLE 180

 Query: 181 FEATQCPNPFSYVPRPLSSHSDHMTFLQRVKNMLIAFSQNFLCDVVYSPYATLASEFLQR 240
              EATQCPNPFSYVPRPLS+HSDHMTFLQRVKNMLIAFSQNFLCDVVYSPYATLASEFLQR
 Sbjct: 181 SEATQCPNPFSYVPRPLSAHSDHMTFLQRVKNMLIAFSQNFLCDVVYSPYATLASEFLQR 240
```

C

Peptide Sequences

peptides	Sequence
peptide 1	VVPLY

Mapping result

peptide 1: VVPLY

Serial number	DetoxiProt ID	Protein name	Species	Start	End	Detail
1	DP00003	ADHX	Caenorhabditis elegans	92	97	view
2	DP01790	ADH	Bombyx mori	91	96	view
3	DP05353	ADH3	Strongylocentrotus purpuratus	92	97	view

D

Search by transmembrane_domain

Display 15 items per page Go!

Page 1 Total Page:109 Total amount:1621

first page | previous page | next page | last page

DetoxiProt ID	Name	Species	classification	Phase	Detail
DP00014	CYP-35D1	Caenorhabditis elegans	Cytochrome P450	Phase I	view
DP00015	FMO-1	Caenorhabditis elegans	Flavin-containing MonoOxygenase	Phase I	view
DP00016	FMO-2	Caenorhabditis elegans	Flavin-containing MonoOxygenase	Phase I	view
DP00018	FMO-4	Caenorhabditis elegans	Flavin-containing MonoOxygenase	Phase I	view
DP00034	HSD-1	Caenorhabditis elegans	The short-chain dehydrogenase/reductase	Phase I	view
DP00035	HSD-2	Caenorhabditis elegans	The short-chain dehydrogenase/reductase	Phase I	view

E

3D-Blast in Database

3D visualization for the query protein:

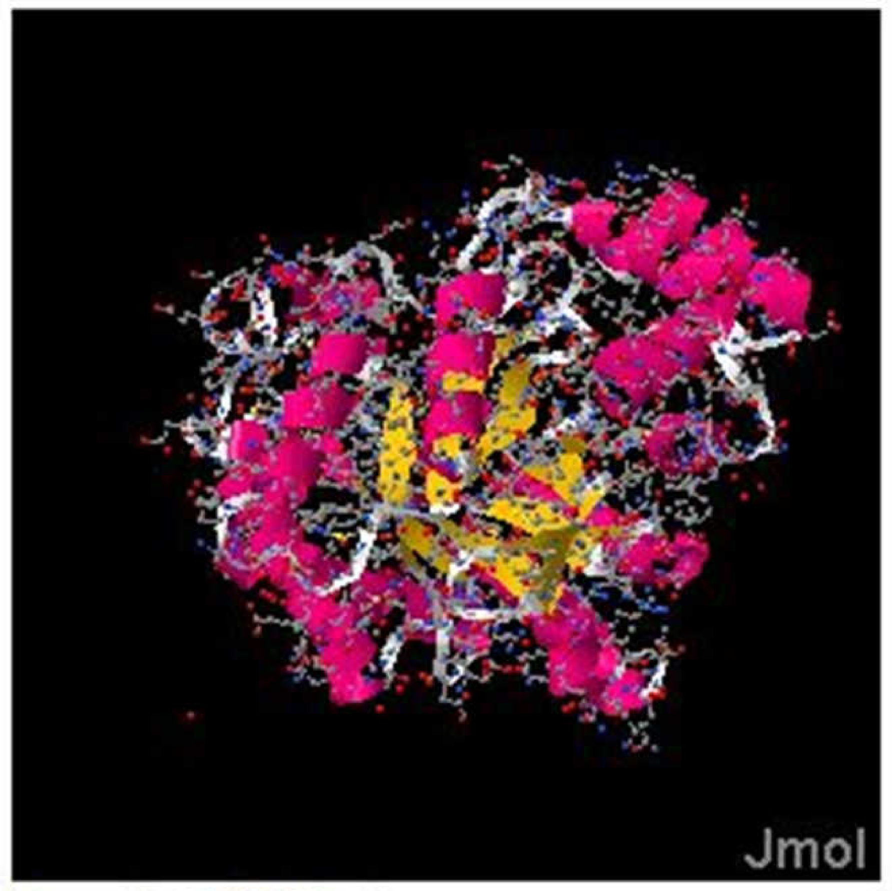

atoms 20% 100%

Reset to original orientation

spin

Rotate about the x axis

background black

```
                                                                  Score     E
Sequences producing significant alignments:                       (bits)  Value

2is7_A CRYSTAL STRUCTURE OF ALDOSE REDUCTASE COMPLEXED WITH DICH...   739     0.0
2acr_A AN ANION BINDING SITE IN HUMAN ALDOSE REDUCTASE: MECHANIS...   674     0.0
1ads_A AN UNLIKELY SUGAR SUBSTRATE SITE IN THE 1.65 ANGSTROMS ST...   672     0.0
2ine_A CRYSTAL STRUCTURE OF ALDOSE REDUCTASE COMPLEXED WITH PHEN...   671     0.0
3g5e_A HUMAN ALDOSE REDUCTASE COMPLEXED WITH IDD 740 INHIBITOR        664     0.0
2iq0_A CRYSTAL STRUCTURE OF ALDOSE REDUCTASE COMPLEXED WITH HEXA...   664     0.0

 >2ine_A CRYSTAL STRUCTURE OF ALDOSE REDUCTASE COMPLEXED WITH PHENYLACETIC
         ACID
        Length = 310

  Score = 671 bits (1752), Expect = 0.0
  Identities = 271/310 (87%), Positives = 304/310 (98%)

 Query: 2    KHKISQTEKFMPKKEXVQTFCLLMACACACDYBSRHXPFHEKDGSQKMDCBBDCYBACYC  61
              KHKISQTEKFMPKKEXVQTFC+LMACACACDY+SRHXPFHEKDGSQKMDCBBD++BACY+
 Sbjct: 1    KHKISQTEKFMPKKEXVQTFCDLMACACACDYCSRHXPFHEKDGSQKMDCBBDYCBACYB  60

 Query: 62   SRVPKIBQPEEHFFKKIBRXSCDQGCDYYBYCYYASRPVPFVPEFNVTQPEKTXTQPMSE  121
              S+VPK+BQPEEHF+KKIBRX  C++GCD++BY+++ASRPVPFVPEFNVTQPEKTXTQP  SE
 Sbjct: 61   SQVPKCBQPEEHFKKKIBRXTCCGGCDCBBYBACASRPVPFVPEFNVTQPEKTXTQPDSE  120

 Query: 122  FKLWMTNNFFQPKCDYBBDBBBGDCCSQVPVPHKEVQTKIBYCBACAQTVPHMPKNXPHH  181
              FK++ T++FFQPKCDYBB+B++GD++SQVPVPHKEVQ KI+YCBACAQTV HMPKNXPHH
 Sbjct: 121  FKISQTKKFFQPKCDYBBBBDGGDBYSQVPVPHKEVQKKIDYCBACAQTVQHMPKNXPHH  180
```
