# Supplementary material for: DetoxiProt: an integrated database for detoxification proteins
Source: BMC Genomics. 2011 Nov 30;12(Suppl 3):S2. doi: 10.1186/1471-2164-12-S3-S2 (PMC3333179; doi:10.1186/1471-2164-12-S3-S2)

Phylogenetic tree for Cytosolic sulfotransferase of Mus musculus

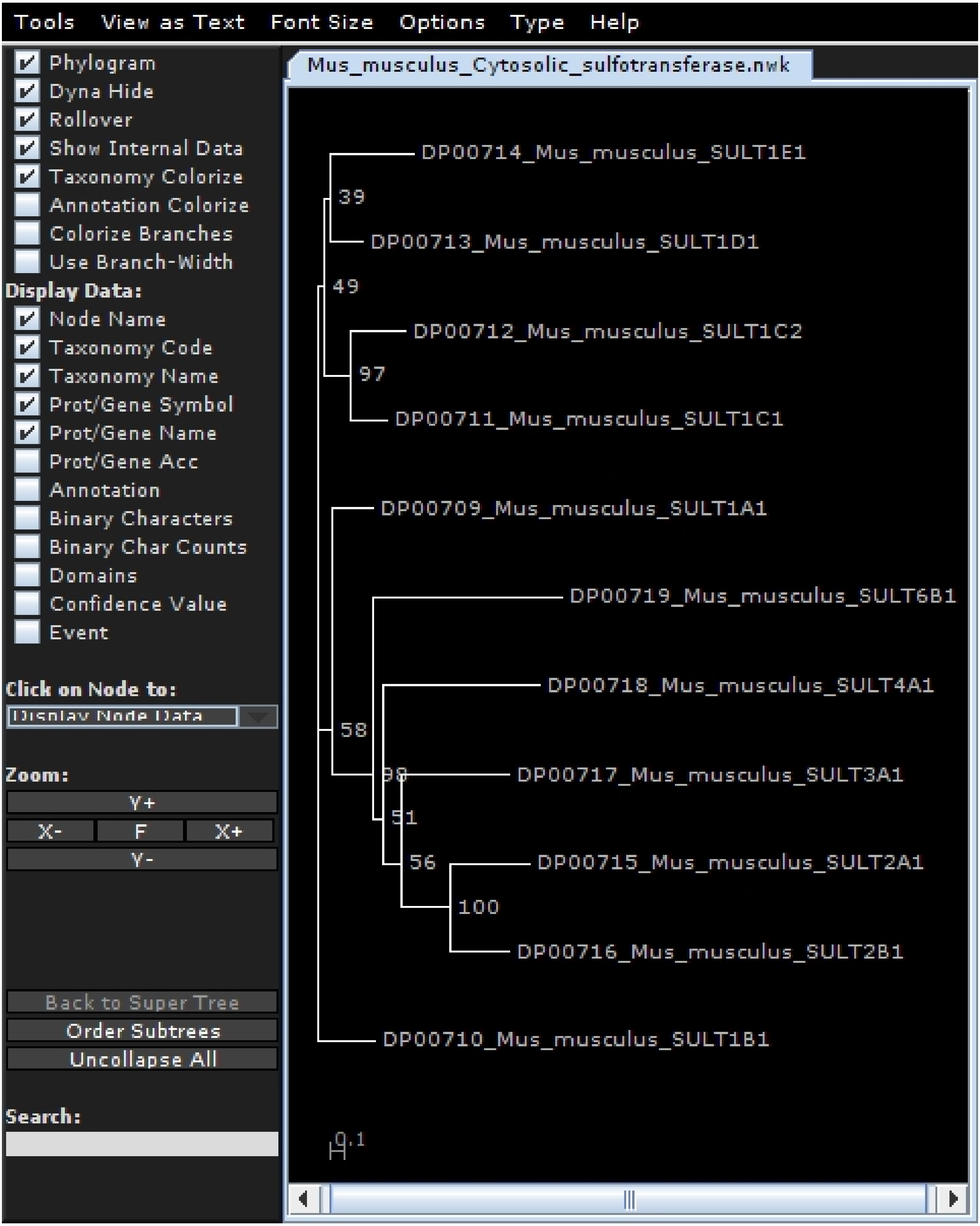

Supplement: Additional file 3 — Illustration of the phylogentic tree of Cytosolic sulfotransferases of Mus musculus by the Archaeopteryx tree viewer. Multiple Sequence Alignment was performed by MUSCLE, phylogenetic tree was generated by Maximum Likelihood (ML) method embedded in RAxML. The nonparametric bootstrap test was performed for 100 replicates. [file 1471-2164-12-S3-S2-S3.pdf]
