# Supplementary material for: DetoxiProt: an integrated database for detoxification proteins
Source: BMC Genomics. 2011 Nov 30;12(Suppl 3):S2. doi: 10.1186/1471-2164-12-S3-S2 (PMC3333179; doi:10.1186/1471-2164-12-S3-S2)
Supplement: Additional file 4 — Phylogenetic distribution of detoxification proteins in model organisms. A relative small number of protein families were found in invertebrates than that in vertebrates. [file 1471-2164-12-S3-S2-S4.pdf]

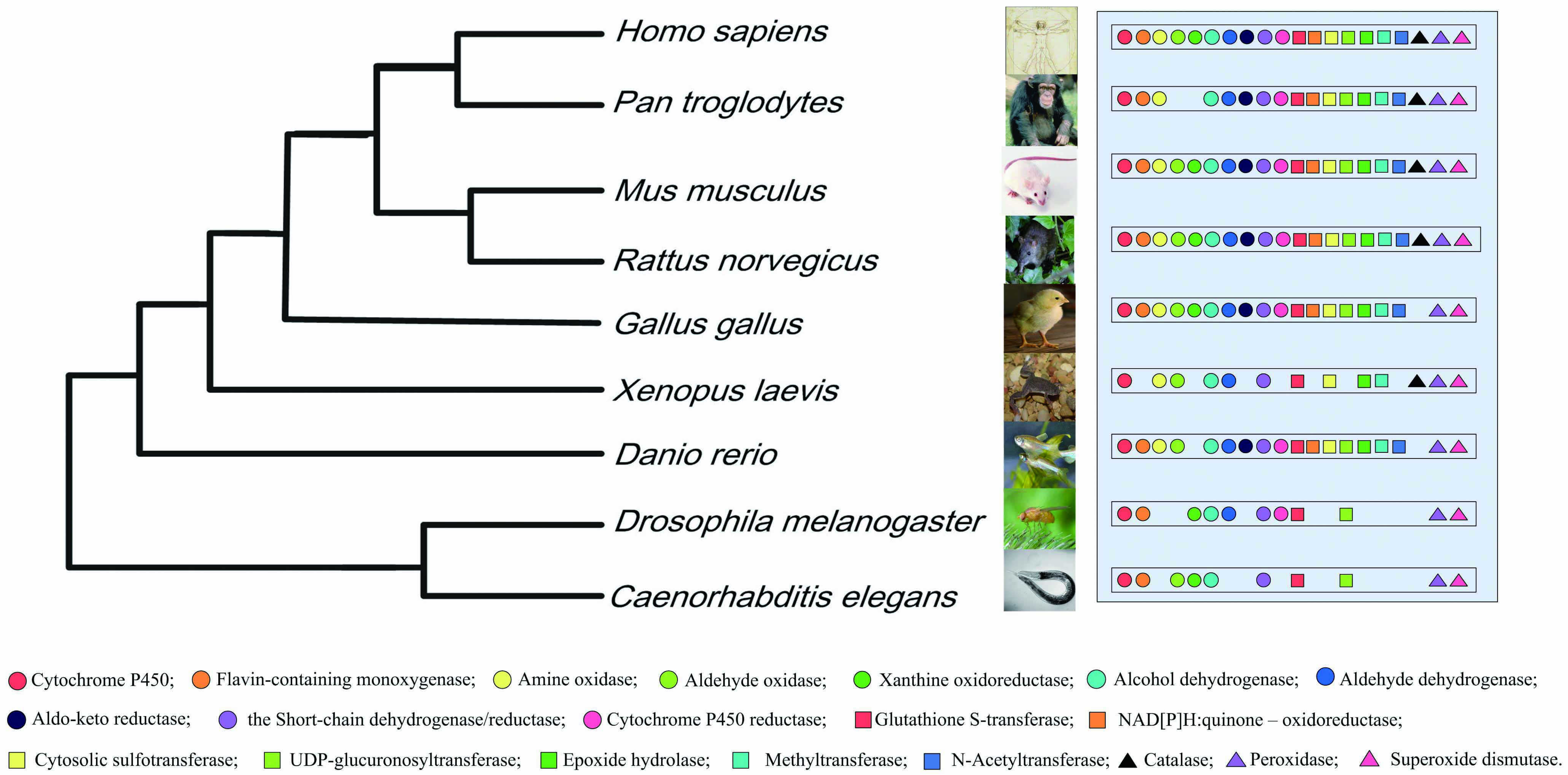

Homo sapiens
Pan troglodytes
Mus musculus
Rattus norvegicus
Gallus gallus
Xenopus laevis
Danio rerio
Drosophila melanogaster
Caenorhabditis elegans
Cytochrome P450;
Flavin-containing monoxygenase;
Amine oxidase;
Aldehyde oxidase;
Xanthine oxidoreductase;
Alcohol dehydrogenase;
Aldehyde dehydrogenase;
Aldo-keto reductase;
the Short-chain dehydrogenase/reductase;
Cytochrome P450 reductase;
Glutathione S-transferase;
NAD[P]H:quinone – oxidoreductase;
Cytosolic sulfotransferase;
UDP-glucuronosyltransferase;
Epoxide hydrolase;
Methyltransferase;
N-Acetyltransferase;
Catalase;
Peroxidase;
Superoxide dismutase.
