# Supplementary material for: DetoxiProt: an integrated database for detoxification proteins
Source: BMC Genomics. 2011 Nov 30;12(Suppl 3):S2. doi: 10.1186/1471-2164-12-S3-S2 (PMC3333179; doi:10.1186/1471-2164-12-S3-S2)

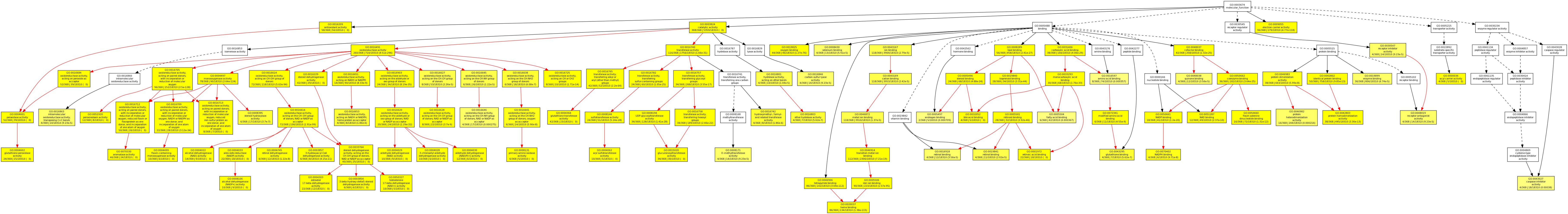

Supplement: Additional file 5 — Gene ontology enrichment analysis of human detoxification genes. This analysis was performed using GOEAST: http://omicslab.genetics.ac.cn/GOEAST/ [file 1471-2164-12-S3-S2-S5.pdf]
